# Investigations on Epoxy-Carbamate Foams Modified with Different Flame Retardants for High-Performance Applications

**DOI:** 10.3390/polym13223893

**Published:** 2021-11-11

**Authors:** Simon T. Kaysser, Christian Bethke, Isabel Fernandez Romero, Angeline Wo Weng Wei, Christian A. Keun, Holger Ruckdäschel, Volker Altstädt

**Affiliations:** 1 Department of Polymer Engineering, University of Bayreuth, Universitaetsstrasse 30, 95447 Bayreuth, Germany; kaysser@comprisetec.de (S.T.K.); christian.bethke@uni-bayreuth.de (C.B.); ruckdaeschel@uni-bayreuth.de (H.R.); 2 CompriseTec GmbH, Rödingsmarkt 20, 20459 Hamburg, Germany; isabelfernandezromero16@gmail.com (I.F.R.); angelinewoww@gmail.com (A.W.W.W.); keun@comprisetec.de (C.A.K.); 3 Bavarian Polymer Institute, University of Bayreuth, Universitaetsstrasse 30, 95447 Bayreuth, Germany; 4 Bayreuth Institute of Macromolecular Research, University of Bayreuth, Universitaetsstrasse 30, 95447 Bayreuth, Germany

**Keywords:** epoxy foam, carbamate, blowing agent, latent curing agent, flame retardance

## Abstract

In transport sectors such as aviation, automotive and railway, materials combining a high lightweight potential with high flame retardant properties are in demand. Polymeric foams are suitable materials as they are lightweight, but often have high flammability. This study focuses on the influence of different flame retardants on the burning behavior of Novolac based epoxy foams using Isophorone Diamine carbamate (B-IPDA) as dual functional curing and blowing agent. The flame retardant properties and possible modifications of these foams are systematically investigated. Multiple flame retardants, representing different flame retardant mechanisms, are used and the effects on the burning behavior as well as mechanical and thermal properties are evaluated. Ammonium polyphosphate (APP), used with a filler degree of 20 wt.% or higher, functions as the best performing flame retardant in this study.

## 1. Introduction

For lightweight applications, polymer foams are in high demand. They are suitable as insulation material, but also as core material for load bearing sandwich constructions. For a wide range of applications, e.g., aircraft interiors, electronics and electric cars, flame retardant properties are an essential requirement. New production techniques for sandwich parts, such as sandwich sheet molding compound (SMC), demand foam core materials to fulfill special requirements during processing. The material has to exhibit sufficient compressive stiffness and strength to withstand more than 25 bar of processing pressure, as well as temperatures up to 130–140 °C in order to obtain an applicable sandwich production process and sufficient part quality [1,2,3].

Common thermoplastic foam cores are limited by the required combination of mechanical and thermal performance. Thus, high temperature resistant foams with adequate compression stiffness and strength are desired, which allow higher process temperatures and pressures, resulting in optimized process times and part quality. In particular, carbon fiber SMC (cSMC) for high performance parts and lightweight design requires higher molding pressures compared to standard glass fiber SMC.

Rigid epoxy foams are a suitable solution due to their outstanding mechanical, thermal and chemical resistance, as well as its electrical insulation properties. They are applied in demanding applications in electronics, marine, aviation and space industry [4,5,6,7,8,9,10]. Most epoxy foams are produced nowadays using hazardous or environmentally harmful chemical blowing agents, partly already listed in SNAP or REACH regulations. The use of physical blowing agents for the production of epoxy foams, such as supercritical CO_2_, is challenging due to the step-polymerization kinetics of epoxy systems, resulting in inferior morphologies and properties [11].

Therefore, the epoxy foaming approach using CO_2_-blocked amine hardeners, so called carbamates, as dual functional curing and blowing agents, received a lot of attention in recent years. They provide a potential processing approach for high performance epoxy foams without using additional hazardous chemical blowing agents. Ren et al. and Bethke et al. focused on several common amine curing agents for carbamate synthesis such as N-Aminoethylpiperazine (AEP), m-Xylenediamine (mXDA), 4,4′Diaminodicyclohexyl-methane (DDCM) and 4-Methylcyclohexane-1,3-Diamin (DMC). At elevated temperature, the carbamate salt decomposes to release the active amine curing agent and CO_2_, resulting in a simultaneous foaming and curing reaction [6,12,13,14]. In a previous study, Isophoronediamine (IPDA) as a new feasible amine curing agent for the carbamate foaming approach was already introduced [15].

The results of Takigushi et al., Lyu et al. as well as Bethke et al. regarding epoxy foams using physical or chemical blowing agents have shown the influence of rheology on the morphology of epoxy foams. It was shown that an increase of matrix viscosity via pre-curing results in a finer and more homogeneous foam morphology, caused by an improved stabilization of the foam cells and decreased CO_2_ diffusion [5,11,16]. Wang et al. have presented limited foaming as an approach to produce microcellular epoxy foams with chemical blowing agents, showing that, by means of closed molds, a much higher control of foam morphology and smaller cell sizes can be achieved compared to free foaming [17]. Chen et al. have shown the nucleating effect of SiO_2_ particles, when added in epoxy foams, leading to an increased number of cells per volume and a decreased average cell size [18].

Applications in aircraft and railway interior as well as for electronics demand enhanced FST (Fire, Smoke, Toxicity). properties. Aircraft interior components have to fulfill strict FST-regulations according to FAR 25.853 [19,20,21]. For most electronic and other applications, a rating of UL94 V-0 is necessary. The combustion process of polymers can be retarded by physical or chemical means. Physical means will vary physical parameters such as decreasing the overall temperature, lowering the concentration of the combusting material or by creating a protective layer between the combusting material and the flame. On the other hand, a chemical flame retardant interferes with the radicals created during the combustion process, impeding their proliferation [22].

Most flame retardants (FR) have specified process and service temperatures, so, when considering curing temperatures of around 160 °C and current EU regulations restricting halogen flame retardants, the available flame retardants are reduced to four established types: aluminum tryhydroxide (ATH), ammonium polyphosphate (APP), 9,10-dihydro-9-oxa-10phosphaphenantherene-10-oxide (DOPO) and melamine phosphate (MP) [23]. The effects and interactions of these flame retardants will be under review and investigation throughout this paper. ATH is a mineral hydroxide with physical interactions, which are characterized by their natural high burning temperature and their thermal degradation, producing a non-combustible char material and water as a by-product [24]. On the other hand, APP, DOPO and MP are chemically interacting and belong to the category of phosphorous containing compounds. They are further classified into organic and inorganic compounds. Organophosphorus compounds such as DOPO may transform to phosphoric acid and then to poly (phosphoric acid) due to constant exposure to a heat source. In addition, their active hydrogen can react with a variety of electron-deficient derivative, which results in a range of phosphaphenanthrene skeleton [25]. APP decomposes on two well-known steps. In the first one, between 290 °C and 450 °C, condensation and cross-linking of the polyphosphate chains to poly (phosphoric acid) takes place releasing NH_3_ and water. Afterwards, at even higher temperatures, the cross-linked poly (phosphoric acid) evaporates or sublimate due to further dehydration [26]. MP decomposes into phosphoric acid, which can carbonize and produce a dense char barrier. The nitrogen bonded to the triazine groups in the melamine site helps to insulate the material from the fire in the gaseous phase [27].

For compact compounds, the interaction between the additives and the matrix can be a matter of polarity and surface energy of the particle itself. When considering foams, the effect of the FR on the morphology of the foam itself and it influence on the mechanical and thermal properties has to be considered next to the burning behavior [28,29].

To the best knowledge of the authors, the effects of different flame retardants in a carbamate foamed epoxy matrix has been rarely reported yet. Therefore, this report focuses on the evaluation of different flame retardants, namely ATH, APP, DOPO and MP for rigid high-performance epoxy foams using carbamates as blowing agents. The goal of the presented study is the development of an epoxy-carbamate foam formulation which passes the UL94 HB criteria with the lowest possible flame-off time, low density and high compressive strength for further development of high-performance applications in aviation and other industries [30].

Therefore, a base system of Epoxy Novolac resin (EN) with CO_2_-blocked Isophorone Diamine (B-IPDA) with a fixed neat density of 0.3 g/cm^3^ and the process approach, developed in a former study was used and modified with a range of commercially available flame retardant additives, such as different grades of Aluminum Hydroxide (ATH), Ammonium Polyphosphate (APP) Dihydrooxaphosphaphenanthrene (DOPO) and Melamine Phosphate (MP) [15]. For each flame retardant a singular filling study was performed. The resulting foam properties and their burning behavior were evaluated and compared.

## 2. Materials and Methods

### 2.1. Materials

Epoxy novolac (EN, DEN431, Olin, MO, USA) was used as received with an epoxy equivalent weight (EEW) of 173.5 g/moL, and a pre-determined viscosity of 19 Pa s (25 °C). In this study, Isophorone Diamine (IPDA) was used in the CO_2_-blocked form as carbamate salt. The blocking reaction of the amine curing agent was conducted according to Ren et al. and Bethke et al. [13,14,15]. Isophorone Diamine (IPDA) with a molecular weight (Mw) of 170.3 g/moL and an amino hydrogen equivalent weight (AHEW) of 42.6 g/moL, (Aradur 22962, Huntsman, TX, USA) was used.

As flame retardants, four different base types were chosen with specific modifications: (a) aluminum hydroxide (ATH), (b) Ammonium Polyphosphate (APP), (c) Melamine Polyphosphate (MPP) and (d) Dihydrooxaphosphaphenanthrene (DOPO) in form of granulate (NOFIA). The materials were used as received. The specifications are listed in Table 1.

Three small aluminum molds with a cavity of 30 × 30 × 10 mm^3^ and a bulk volume of 9 cm^3^ each were applied, produced inhouse at CompriseTec GmbH (Hamburg, Germany). As mold release agent, Loctite Frekote 770-NC (Henkel, Duesseldorf, Germany) was used.

### 2.2. Experimental Procedure

#### 2.2.1. Dispersion

At first, the agglomerated B-IPDA particles were crushed using a simple metal roller and a PE-bag to ensure a proper processing. The B-IPDA/epoxy resin mixture was mixed in a stoichiometric ratio of 76.6/23.4 wt.% and pre-mixed by hand with a wooden spatula at 40 °C. For proper dispersion, a three-roll mill setup 80 E Plus with SiC rollers (EXAKT Advanced Technologies GmbH, Norderstedt, Germany) was used. The parameters for the three-roll mill were set to 50 °C roller temperature with a rotational speed of 200 rpm and gap sizes of 60/20 µm for the first and 20/5 µm for the second run. After dispersion, the batches were stored at −30 °C. Before use, the batches were pre-heated at 40 °C and the flame retardants were dispersed using an overhead stirrer.

#### 2.2.2. Foaming and Specimen Preparation

**Figure 1:**
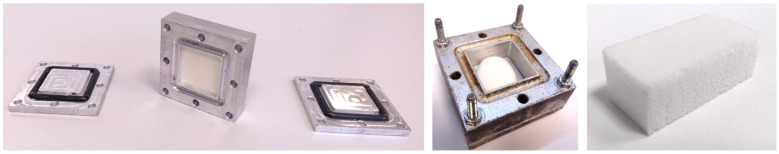
**Left**: small aluminum molds with a cavity of 30 × 30 × 10 mm^3^, **Middle**: mold filled with foam formulation, **Right**: halved foam specimen.

The specimens for compression testing, thermal analysis, horizontal burn tests and morphology investigations were foamed in the small aluminum molds, which were treated with release agent and dried at room temperature for 10 min before use. A specific amount of 2.8 g—equal to a neat foam density of 0.3 g/cm^3^—plus added fillers of the dispersed resin-carbamate-flame retardant mixture was introduced into each mold. The closed molds were transferred into a laboratory oven UFE 500 (Memmert GmbH + Co. KG, Schwabach, Germany), which was preheated and set at 160 °C, for 90 min for foaming and curing. Figure 1 shows the molds used in this work. The specimens for testing were cut using a band saw (Fa. Metabowerke GmbH, Germany). For each specimen series, at least three specimens were produced for comparison.

#### 2.2.3. Characterization Methods

The density of the specimens was measured using a laboratory scale and caliper. The glass transition temperature (T_g_) was determined using dynamic mechanical analysis (DMA) with a temperature range of 30–200 °C, using a Rheometric Scientific RDA III (Rheoservice GmbH & Co. KG, Reichelsheim, Germany).

The optical characterization of the foam morphology for initial trials was carried out using a digital microscope. For main trials, the characterization of the foam morphology was carried out via SEM with a JSM-6510 (Jeol, Akishima, Tokyo, Japan). Samples were sputtered with a 13 nm gold layer (99.9% purity) by using a Sputter Coater 108auto (Cressington, Waltford, England).

The evaluation of cell size and cell size distribution was carried out using the software ImageJ.

Mechanical compression tests were carried out using a universal testing machine ZMART PRO 1455 20 kN (ZwickRoell, Ulm, Germany), referring to DIN EN ISO 844:2014-11 with a preload of 1 N and a test speed of 1 mm/min up to a deformation of 70% [34].

Burning tests were carried out by a horizontal burn test referring to UL94 with a reduced specimen size of 29 × 13 × 9 mm^3^. Thus, only the burning behavior up to the 25 mm-mark could be evaluated. Since most of the modified specimens did not exhibit a flame spread up to the 25 mm-mark, the results can be seen as valid for an approved UL 94 HB rating.

## 3. Results

In this chapter, the effects of different types and filler degrees of selected common flame retardants on the properties of epoxy foams are investigated.

### 3.1. Fill Study of ATH in Epoxy Foams

In a former study, the influence of Aluminum Hydroxide (ATH) as flame retardant for carbamate-foamed epoxy rigid foams was investigated using non-surface modified ATH grades with comparable particle sizes as presented in this study. It was concluded that a high filler degree (up to 50 wt.%) of ATH in an EN-B-IPDA foam leads to a shortened time of extinction. Dripping could also be prevented with the modification of ATH. Therefore, 30–40 wt.% filler degree of ATH were sufficient to reach UL94 HB criteria. The high filler degrees of 40–50 wt.% were necessary to reach flame-off times of below 30 s. However, they resulted in an increased viscosity, leading to poor processability without particle surface modification, as well as an increased density of up to 0.6 g/cm^3^ [15].

Therefore, surface modified grades of ATH were used in this study to evaluate its capability to enhance the dispersibility. The effects on morphology, mechanical properties and burning behavior were evaluated. Table 2 summarizes the specimens prepared for the ATH filling study including the filler degree (Fd), density, average foam cell diameter (d_cell_), compression modulus (E_c_), compressive strength (C_s_) and T_g_, derived from DMA (Peak tan δ).

#### 3.1.1. Morphology of ATH-Modified Epoxy Foams

**Figure 2:**
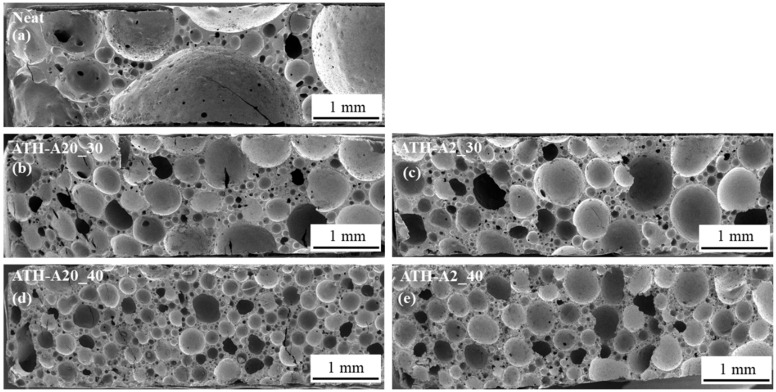
SEM images taken from EN/B-IPDA foams without particle modification, Neat (**a**), with 30 wt.% ATH (**b**,**c**) and 40 wt.% ATH of different particle size A20 (**d**) and A2 (**e**).

The overview SEM images of foam specimens with different ATH particle sizes and filler degrees are presented in Figure 2.

**Figure 3:**
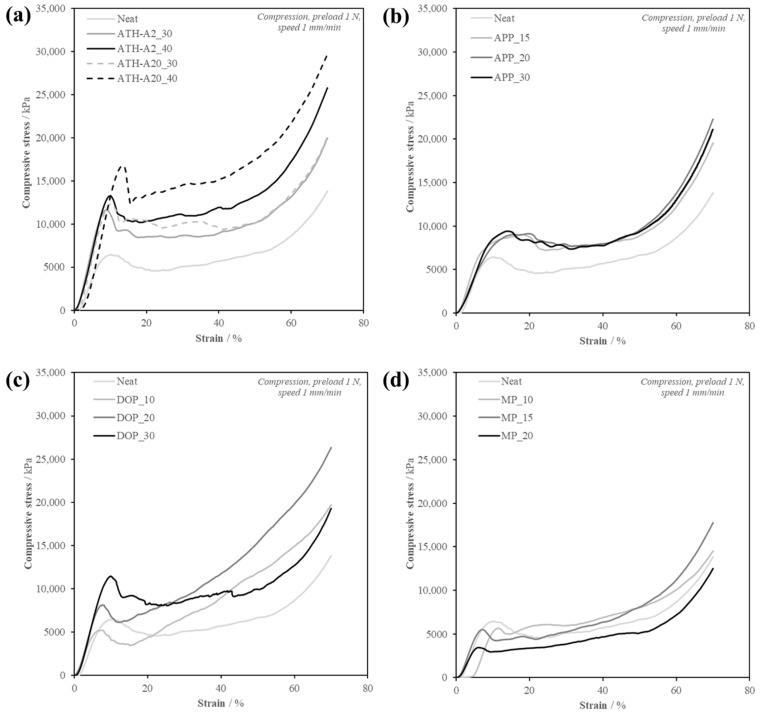
Compression curves of EP foams modified with ATH (**a**), APP (**b**), DOPO (**c**) and MP (**d**).

The neat foam has irregular morphology and lower foam cell density compared to ATH-filled foams. When ATH particles are introduced into the foam system, the foam cell density increases by comparing the neat foam in Figure 3a with ATH foams in Figure 2b to Figure 2e. The particles act as heterogeneous nucleating agents to promote bubble nucleation during the foaming process. When the ATH filler degree increases from 30 wt.% to 40 wt.%, the foam cell density increases, and the foams have relatively smaller foam cell size as can be seen by the comparison of Figure 2b with Figure 2d and Figure 3c with Figure 2e. Foams filled with larger particle size (A2) show a larger foam cell size and less homogeneous morphology. In contrast, foams filled with smaller particle size (A20) exhibit smaller foam cell size and higher foam cell density, leading to a superior compression performance.

#### 3.1.2. Evaluation on the Mechanical and Thermal Properties of ATH-Modified Epoxy Foams

Comparing the thermal properties, the foams modified with ATH-A2 exhibit a lower T_g_ with higher filling degree while ATH-A20 increase the T_g_ slightly. This may be due to a combination of high filler degree and bigger particle size, resulting in a larger foam cell size with the mechanical (DMA) test method. The compression stress–strain curve is divided into three different regions, as seen in Figure 3.

Most of the samples (except Neat) show an elastic behavior region within 5% compressive strain. The neat foam shows the lowest compressive strength and compressive modulus compared to ATH particles-filled foams. In the plateau region, the 40 wt.% ATH modified filled foams exhibit the highest plateau stress, which is resulting from the collapse of the foam cell walls leading to bulk foam fracture. The transition point from plateau region to densification region occurs between 30 and 50% strain. For the neat epoxy foam, the transition point occurs at lower strain percentage, which means it requires lower energy for the adjacent foam cell wall to completely collapse. Particle-filled foams have relatively higher plateau stress and densification stress due to higher foam cell density, smaller cell size and the reinforcing effect of the particles in the matrix.

The epoxy foams filled with ATH of smaller particle size have higher compressive strength, plateau stress and densification stress, but similar bulk foam density. This indicates that the particle size for ATH has more influence on the compression properties when comparing a similar bulk foam density. With smaller particle size, more particle surfaces and surface area are available for particle–matrix interaction. Although, it has to be considered that smaller particles tend more towards agglomeration and require a better dispersion. As it can be derived from Table 2, ATH A-20 modified foams show increased compression strength (12.3 MPa) compared to foams using non-surface modified ATH-S30 (WTH, Reflamal S30, Fd 30 wt.%, C_s_ 11.2 MPa) used in the former study. A slight decrease in T_g_ can be observed from using ATH without (157 °C) and using ATH with surface modification [15].

#### 3.1.3. Flame Retardance of ATH-Modified Epoxy Foams

**Figure 4:**
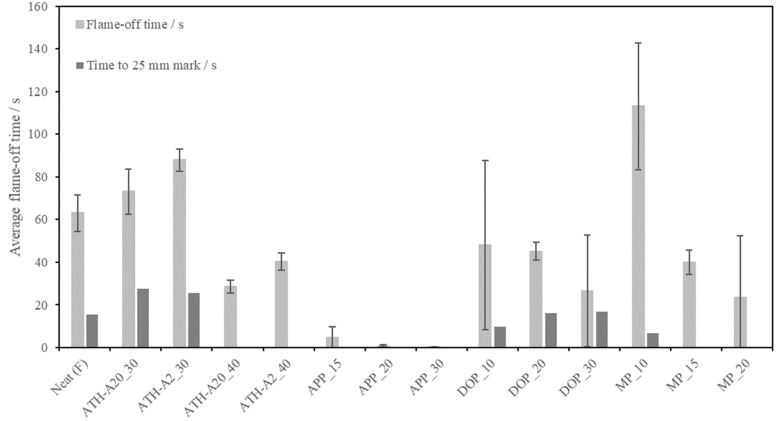
Average flame-off time of flame retardant modified EN/B-IPDA foam specimens ref. to UL94 Horizontal Burn (from left to right: neat, ATH, APP, DOPO, MP); specimens marked with (F) failed the UL94 HB criteria.

Figure 4 shows the comparison of the flame off time of samples in the horizontal burn setup. It is noted that the times to 25 mm mark are displayed for those specimens with a flame propagation as far as the 25 mm mark only.

It can be observed that with higher ATH filler degree the burning time decrease. More ATH particles release a higher amount of water and form a thicker oxide layer during thermal decomposition, thus the flame extinguished in a shorter time.

ATH-A20 modified foams also show shorter flame-off times, higher compressive strength, plateau stress and densification stress with similar bulk foam density, comparing to the bigger ATH-A2 particles. In addition, they show comparable flame retardant performance to non-surface modified ATH particles used in a former study (WTH, Reflamal S2, Fd 40 wt.%, 34 s extinction time), but with improved processability [15]. Thus, the ATH-A20 particles will be used for future studies.

### 3.2. Fill Study of APP in Epoxy Foams

Since APP is mainly active in the gas phase, it is known for an effective flame protection for porous materials [35]. In this study, APP modifications of the epoxy foams were evaluated with different filler degrees. Table 3 summarizes the tested formulations with their density and T_g_, derived from DMA (Peak tan δ).

#### 3.2.1. Morphology of APP-Modified Epoxy Foams

**Figure 5:**
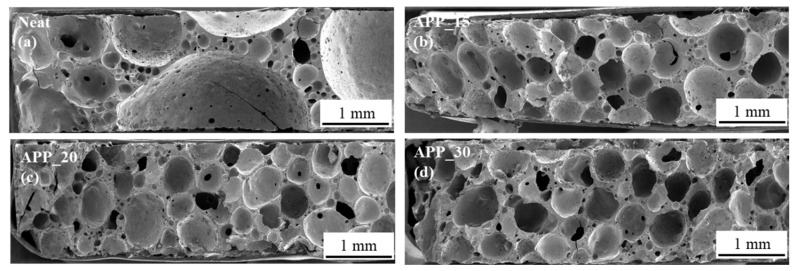
SEM images taken von EN/B-IPDA foams without particle modification, Neat (**a**), with APP 15 wt.% (**b**), 20 wt.% (**c**), 30 wt.% (**d**).

As it can be derived from the SEM images in Figure 5b–d, the epoxy foams filled with APP particles exhibit a morphology with only slightly higher foam cell density, smaller cell size and more homogeneous as the filler degree increases from 15 wt.% to 30 wt.%. The effect is much less significant than when using ATH (Figure 2).

#### 3.2.2. Mechanical and Thermal Properties of APP-Modified Epoxy Foams

As it can be seen in Table 3, the modification with APP slightly increases the T_g_ derived from DMA (Peak tan δ), probably due to the more homogeneous foam morphology and its effects on the specimen’s mechanical properties. The mechanical compression properties were evaluated and shown in Figure 3.

It is shown that with introducing APP the compression properties improve compared to the neat foam. Although, it has to be considered that the density is also increased. In general, 30 wt.% APP modified epoxy foams exhibit a slightly better compression performance. The explanation is analogue to the ATH-filled foams, as they are both mainly stiff spherical particles of similar size and working as nucleation sides.

#### 3.2.3. Flame Retardance of APP-Modified Epoxy Foams

As the APP particle filler degree increases, the flame off time decreases to a very low level compared to ATH-modified foams as seen in Figure 4. No dripping could be observed. All APP-modified specimen passed the UL94 horizontal burn criteria.

In conclusion, the compression properties and flame retardant performance are improved when the APP filler degree increased.

### 3.3. Fill Study of DOPO in Epoxy Foams

Organophosphorous compounds like DOPO function mainly as radical scavengers in the gas phase [36].

#### 3.3.1. Morphology of DOPO-Modified Epoxy Foams

**Figure 6:**
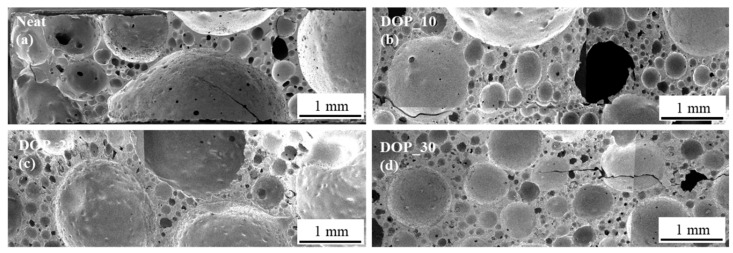
SEM images taken von EN/B-IPDA foams without particle modification, Neat (**a**), with DOPO 10 wt.% (**b**), 20 wt.% (**c**), 30 wt.% (**d**).

An initial morphology study showed that, despite of the DOPO concentration that the modification with DOPO leads to a strongly bimodal cell morphology as shown in Figure 6. This also shows in the large standard deviation of the average cell diameter (Table 4). The addition of a filler degree of 20 wt.% or higher resulted on comparable foam cell distribution.

The irregular cell morphology of the DOPO modified specimens with a small number of big cells resulted in very large standard deviations of the trials and low reproducibility.

#### 3.3.2. Mechanical and Thermal Properties of DOPO-Modified Epoxy Foams

Table 4 summarizes the tested formulations with their density and T_g_, derived from DMA (Peak tan δ). First, the difficulty found when handling DOPO containing compounds must be addressed. Using a concentration of 40 wt.% or higher increased the viscosity notably and disturbed the overall processability. In addition, an increase in viscosity could be seen with the DOPO modified batches, even when stored at temperatures of −30 °C over time. This effect was not observed with other compositions used in this study. This behavior can be caused by the entanglement of the DOPO molecules with the epoxy molecules, which could not be reversed during the pre-heating phase at 40 °C. Another possibility is the reaction of DOPO with epoxy molecules, causing an increased molecular weight resulting in an increased viscosity, a decrease in the molecular network density due to loss of connection points, and a lower T_g_. The results of the compression tests presented in Table 4, show a clear improvement of the compressive strength when the DOPO filler degree was increased. With increasing DOPO concentration, the plastic region of the curve was augmented as the presence of the solid particles helped to strengthen the cell wall, similar to former results using ATH and APP. A remarkable increase of the compressive strength and modulus was observed with DOP_30, due to the ability of the matrix to transfer the load to the solid additive. However, the compressive tests with 30 wt.% DOPO-modified foam result in a higher standard deviation compared to for example APP modified foams.

#### 3.3.3. Flame Retardance of DOPO-Modified Epoxy Foams

It is observed from Figure 4, that with increasing the filler degree, the flame off time decreased. This was expected as a higher amount of DOPO molecules release more radical scavengers and therefore suppress the radicals which could propagate the flame. In addition, DOPO lead to the formation of a char layer, shielding the material from the flame saving the core of the sample. However, all three DOPO modified epoxy foams show dripping and therefore fail the UL94 horizontal burn criteria. In addition, the DOPO modified specimens are showing high standard deviations in flame off time. This can be caused by an inhomogeneous distribution of flame retardance due to the difficult processability of the DOPO modified systems.

From these results it was concluded that the optimal filler degree of DOPO is 30 wt.%, leading to the best compressive properties and the fastest flame off times.

### 3.4. Fill Study of Melamine Phosphate in Epoxy Foams

Melamine phosphate (MP) is also known for its radical scavenger effects while decomposition. In addition, melamine compounds are known to show positive effects with other flame retardants [36,37]. Table 5 summarizes the tested formulations with their density as well as morphology and compression properties.

#### 3.4.1. Morphology of MP-Modified Epoxy Foams

**Figure 7:**
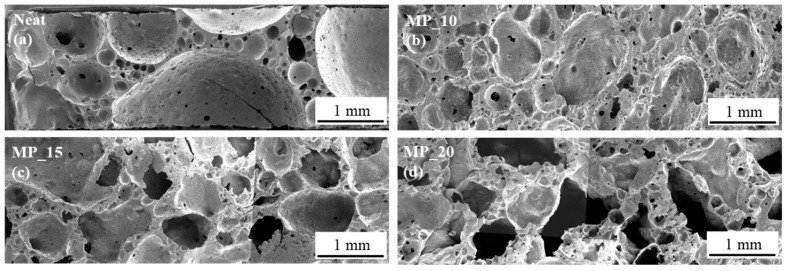
SEM images taken von EN/B-IPDA foams without particle modification, Neat (**a**), with MP 10 wt.% (**b**), 15 wt.% (**c**), 20 wt.% (**d**).

The initial morphology investigation showed that MP led to very inhomogeneous and anisotropic foam cell structures, which increased in size as the concentration of MP increased (Table 5). Figure 7 shows the morphology of MP-modified epoxy foam specimens with increasing MP-filler content.

The anisotropy of the morphology and the cell size increase with the filler degree of MP. In addition, in contrast to ATH, APP or DOPO modified foams, MP modified foams with 15–20 wt.% MP show a significantly irregular cell shape—tending towards an open cell morphology, resulting in lower mechanical properties.

#### 3.4.2. Mechanical and Thermal Properties of MP-Modified Epoxy Foams

Based on the result, that the epoxy foams using a filler degree of 20 wt.% or higher of MP show a semi-rigid behavior, it was decided to investigate the effect of adding max. 20 wt.% to the composition. The T_g_ of the foamed system, as shown in Table 5, is not significantly influenced by the filling with MP.

The mechanical properties as presented in Table 5 and Figure 3d show a decrease of the compressive strength of the samples as a consequence thereof. This is caused by a negative interaction between the filler and the matrix, preventing a proper distribution of the load resulting in an overall decrease of the compressive properties. It can be seen how there is an initial delay of the strain-stress curves. This could be caused by the collapse of the larger foam cells at the beginning of the test, due to their anisotropy the cells that were perpendicular to the load direction would collapse faster compared to spherical cells.

#### 3.4.3. Flame Retardance of MP-Modified Epoxy Foams

Comparing the flame retardance of the MP-modified specimens in Figure 4, it can be seen that the flame-off time decreased as the amount of MP was increased. This behavior was expected, as higher concentrations lead to more active sites that could help to extinguish the flame, resulting in a pass for the UL94 horizontal burn criteria for 15 and 20 wt.% of MP. However, a high standard deviation must be considered. This can be explained by the significant irregular cell morphology and the small specimen size.

Based on these results, it was concluded that the optimal filler degree was of 15 wt.% as it reached a compromise between the decrease on the mechanical properties and the improvement of the flammability properties.

## 4. Summary of Mechanical Properties and Flame Retardance

**Figure 8:**
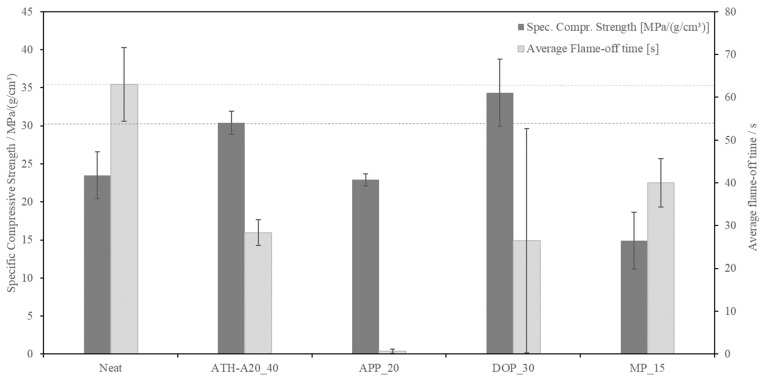
Average specific compressive strength (compressive strength normalized by the foam density), compression tests ref. to DIN EN ISO 844:2014-11, preload 1 N, test speed 1 mm/min, max. deformation 70% (dark grey) and average flame-off time in horizontal burn tests ref. to UL94 with reduced specimen size (light grey).

It is known that the mechanical properties are strongly influenced by the foam density as well as the morphology and the additives working as reinforcing particles. The morphology is strongly influenced also by the type of FR and filler degree. As the target applications require good compressive mechanical performance as well as low density, the (density-)specific compressive strength will be used for comparing these properties in this study. The results of this study are display in Figure 8.

As can be seen, MP as singular FR led to a poor specific compressive strength. MP as FR, as stated before, leads to an irregular and partly open cell morphology. DOPO as a FR, as shown in Section 3.1.3., leads to a bimodal cell morphology with big singular cells but reaches a good specific compressive strength, which can be contributed to DOPO working as reinforcing additive in the matrix. Both samples show high standard deviations for specific compressive strength and flame-off time due to irregular morphology and difficult processing of the FR in the matrix. This could lead to an inhomogeneous FR distribution in the foam. The modification with ATH leads to high compressive strength, but also high density and mediocre flame retardance. ATH is clearly working as reinforcing particle, as already stated in a former study [15]. APP modified foams perform best in the horizontal burn tests by far and reach acceptable mechanical performance with slightly increased density. APP offers the lowest flame-off time, followed by DOPO. This could lead to the conclusion, that FR, which are active in the gas phase are beneficial for low density foams. Although, this must be confirmed via cone calorimeter tests in the future. The inferior flame retardant performance of MP-modified specimens can be contributed to the partly open cell and irregular morphology.

Therefore, and especially with regards to the very low and consistent flame-off time, APP_20 can be selected as superior FR system for future development.

## 5. Conclusions

In this study, a basic epoxy novolac foam, cured and foamed with a dual functional carbamate derived from a reaction of CO_2_ and IPDA amine curing agent (B-IPDA), was used. It was modified using several flame retardants. The systems were analyzed regarding their morphology, density, mechanical and thermal behavior, as well as flammability by UL94 horizontal burn tests. It can be concluded, that APP serves as the best performing singular FR system among the evaluated systems. In addition, it was shown that APP and ATH-modified epoxy foams tend to have a superior foam morphology compared to the other FRs-DOPO and MP—used in this study. Furthermore, the influence of the morphology and density on the mechanical compression performance of the foams was shown. Upcoming work will focus on the combination of flame retardants, their combinatory, synergistic or antagonistic effects and further analysis on bigger specimens, further evaluating the flame-retardant performance.

## Figures and Tables

**Table 1:** Summary of flame retardants used in this study. Particle size derived from technical data sheets [31,32,33].

Type	Trade Name	Supplier/Manufacturer	Specification	Abbreviation
ATH	Addforce FR S20L20	WTH Walter Thieme Handel GmbH	4–5 µm (D50)	ATH-A20
ATH	Addforce FR S2L40	WTH Walter Thieme Handel GmbH	13–18 µm (D50)	ATH-A2
APP	Addforce FR APP 201	WTH Walter Thieme Handel GmbH	<15 µm	APP
MP	Melagard MP	WTH Walter Thieme Handel GmbH/Italmatch Chemicals S.p.A.	4 µm (D50) 25 µm (D98)	MP
DOPO	NOFIA OL3001-P	WTH Walter Thieme Handel GmbH/FRX Polymers (Europe), NV	-	DOPO

**Table 2:** Summary of ATH flame retardants used in this study. Particle size derived from technical data sheets [31,32,33].

Sample	Additive	Fd [wt.%]	Density [g/cm^3^]	T_g_ [°C]	d_cell_ [µm]	E_c_ [MPa]	C_S_ [MPa]
Neat	⎼	⎼	0.3 ± 0.03	157	169 ± 243	123.3 ± 9.3	6.9 ± 1.1
ATH-A20_30	ATH-A20	30	0.43 ± 0.01	153	134 ± 113	180 ± 7	12.3 ± 0.04
ATH-A2_30	ATH-A2	30	0.44 ± 0.02	153	124 ± 113	186 ± 6.2	11.4 ± 0.3
ATH-A20_40	ATH-A20	40	0.51 ± 0.01	155	124 ± 84	190.3 ± 18.9	15.5 ± 1.2
ATH-A2_40	ATH-A2	40	0.51 ± 0.01	149	123 ± 94	184.7 ± 3.5	12.8 ± 0.6

**Table 3:** Specimens of APP fill study: filler degree (Fd), density, T_g_, derived from DMA (Peak tan δ), average foam cell diameter (d_cell_), compression modulus (E_c_), compressive strength (C_s_).

Sample	Additive	Fd [wt.%]	Density [g/cm3]	Tg [°C]	dcell [µm]	Ec [MPa]	CS [MPa]
Neat	⎼	⎼	0.28 ± 0.03	157	169 ± 243	123.3 ± 9.3	6.9 ± 1.1
APP_15	APP	15	0.36 ±0.01	159	148 ± 136	132 ± 9.54	8.84 ± 0.16
APP_20	APP-	20	0.38 ± 0.00	158	155 ± 127	125 ± 17.44	8.77 ± 0.32
APP_30	APP	30	0.44 ± 0.01	157	143 ± 122	135 ± 17.1	9.21 ± 0.45

**Table 4:** Specimens of DOPO fill study incl. comparison specimens: filler degree (Fd), density, T_g_, derived from DMA (Peak tan δ)., average foam cell diameter (d_cell_), compression modulus (E_c_), compressive strength (C_s_).

Sample	Additive	Fd [wt.%]	Density [g/cm^3^]	T_g_ [°C]	d_cell_ [µm]	E_c_ [MPa]	C_S_ [MPa]
Neat	⎼	⎼	0.28 ± 0.03	157	169 ± 243	123 ± 9	6.9 ± 1.1
DOP_10	DOPO	10	0.33 ± 0.02	154	162 ± 238	113 ± 10	4.8 ± 0.4
DOP_20	DOPO	20	0.32 ± 0.01	153	158 ± 210	146 ± 19	8.3 ± 0.9
DOP_30	DOPO	30	0.35 ± 0.03	142	172 ± 181	181 ± 21	11.9 ± 1.5

**Table 5:** Specimens of MP fill study incl. neat comparison specimens: filler degree (Fd), density, T_g_, derived from DMA (Peak tan δ)., average foam cell diameter (d_cell_), compression modulus (E_c_), compressive strength (C_s_).

Sample	Additive	Fd [wt.%]	Density [g/cm^3^]	T_g_ [°C]	d_cell_ [µm]	E_c_ [MPa]	C_S_ [MPa]
Neat	⎼	⎼	0.28 ± 0.03	157	169 ± 243	123.3 ± 9.3	6.9 ± 1.1
MP_10	MP	10	0.35 ± 0.01	157	165 ± 204	113 ± 20	5.1 ± 1.6
MP_15	MP	15	0.36 ± 0.00	156	206 ± 264	115 ± 16	5.3 ± 1.3
MP_20	MP	20	0.38 ± 0.00	157	156 ± 211	86 ± 6	3.0 ± 0.7
